# Melioidosis in a Resident of Texas with No Recent Travel History, United States

**DOI:** 10.3201/eid2606.190975

**Published:** 2020-06

**Authors:** Caitlin M. Cossaboom, Atanaska Marinova-Petkova, Jonathan Strysko, Gretchen Rodriguez, Trevor Maness, Jaime Ocampo, Jay E. Gee, Mindy G. Elrod, Christopher A. Gulvik, Lindy Liu, William A. Bower, Alex R. Hoffmaster, David D. Blaney, Johanna S. Salzer, Jonathan S. Yoder, Mia C. Mattioli, Thomas J. Sidwa, Lillian Ringsdorf, Gale Morrow, Elvia Ledezma, Amanda Kieffer

**Affiliations:** Centers for Disease Control and Prevention, Atlanta, Georgia, USA (C.M. Cossaboom, A. Marinova-Petkova, J. Strysko, J.E. Gee, M.G. Elrod, C.A. Gulvik, L. Liu, W.A. Bower, A.R. Hoffmaster, D.D. Blaney, J.S. Salzer, J.S. Yoder, M.C.Mattioli);; Texas Department of State Health Services, San Antonio, Texas, USA (G. Rodriguez, T. Maness, J. Ocampo, L. Ringsdorf, G. Morrow, E. Ledezma, A. Kieffer);; Texas Department of State Health Services, Austin, Texas, USA (T.J. Sidwa)

**Keywords:** melioidosis, Burkholderia pseudomallei, bacteria, autochthonous, emerging pathogen, endemicity, travel history, zoonoses, Texas, United States, *Suggested citation for this article*: Cossaboom CM, Marinova-Petkova A, Strysko J, Rodriguez G, Maness T, Ocampo J, et al. Melioidosis in a resident of Texas with no recent travel history, United States. Emerg Infect Dis. 2020 Jun [*date cited*]. https://doi.org/10.3201/eid2606.190975

## Abstract

To our knowledge, environmental isolation of *Burkholderia pseudomallei*, the causative agent of melioidosis, from the continental United States has not been reported. We report a case of melioidosis in a Texas resident. Genomic analysis indicated that the isolate groups with *B. pseudomallei* isolates from patients in the same region, suggesting possible endemicity to this region.

*Burkholderia pseudomallei*, which causes melioidosis, is a gram-negative saprophytic bacterium endemic to tropical and subtropical environments worldwide; to our knowledge, isolation from the continental United States has not been reported (*1*–*3*). The most overrepresented risk factor for melioidosis is diabetes mellitus (*3*,*4*). *B. pseudomallei* is resistant to many antimicrobial drugs (*3*). Laboratory exposures might occur without appropriate biosafety precautions (*2*,*5*).

Surveillance is challenging because melioidosis is not nationally notifiable; however, *B. pseudomallei* is a Tier 1 overlap Select Agent (*6*), and the Centers for Disease Control and Prevention (CDC) receives voluntary reports (*2*,*7*). We report a case of melioidosis in a Texas resident who had no recent travel history.

## The Study

On November 17, 2018, a 63-year-old man from Atascosa County, Texas, came to hospital A with fever, chest pain, and dyspnea of 3 days’ duration. At admission, he reported congenital unilateral renal agenesis. Renal function measures were unremarkable. Increased hemoglobin A1c level and hyperglycemia (glucose >200 mg/dL) suggested undiagnosed type 2 diabetes.

Computed tomography of chest and abdomen with intravenous contrast showed left lower lobe pneumonia with a small left pleural effusion. Empiric antimicrobial drug therapy with intravenous azithromycin and ceftriaxone was initiated. Blood culture yielded presumptive *B. pseudomallei*, which was sent for confirmation to the laboratory response network (LRN) site in Houston, Texas.

**Figure 1:**
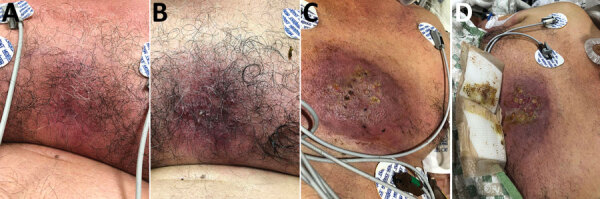
Progressive changes in a cutaneous chest wound for a 63-year-old man who had melioidosis, Texas, USA, 2018. Images were obtained on A) day 3, B) day 4, C) day 9, and D) day 10 after his initial visit to hospital A on November 17, 2018.

On November 20, a localized, violaceous, cutaneous lesion developed on the central anterior chest wall of the patient and progressed to become purulent and ulcerated (Figure 1). The next day, he experienced respiratory failure, was emergently intubated, and was transferred to hospital B.

Hospital B was not aware of the presumptive diagnosis and performed a blood and chest wound culture. Both cultures showed gram-negative rods; blood analysis showed acute kidney injury. On November 25, *B. pseudomallei* susceptible to trimethoprim/sulfamethoxazole and ceftazidime (Table) was identified, and treatment was switched to ceftazidime by using dosing for continuous renal replacement therapy (*4*).

**Table:** Drug susceptibility profile for *Burkholderia pseudomallei* isolate from culture of chest wound from a 63-year-old man, Texas, USA*

Drug	MIC, μg/mL	Result
Amikacin	>32	R
Ampicillin	>16	R
Ampicillin/sulbactam	>16/8	R
Aztreonam	>16	R
Cefazolin	>16	R
Cefepime	>16	R
Cefotaxime	32	R
Cefoxitin	>16	R
Ceftazidime	8	S
Ceftriaxone	>32	R
Cefuroxime	>16	R
Ciprofloxacin	>2	R
Gentamicin	>8	R
Ertapenem	>1	R
Meropenem	<1	S
Piperacillin/tazobactam	<16	S
Tobramycin	>8	R
Trimethoprim/sulfamethoxazole	<2/38	S

*R, resistant; S, susceptible.

On November 26, the patient was extubated and began hemodialysis (3 ×/wk). He was discharged on December 9 and received 3 months of daily trimethoprim/sulfamethoxazole (*4*). Subsequent follow-up showed clinically recovery and resolution of renal insufficiency.

Because the isolate had an unremarkable resistance profile and *B. pseudomallei* was not specifically listed on the Texas Notifiable Conditions List, the automated system at hospital B did not generate an alert indicating it required LRN confirmation, and the isolate was not promptly reported to Texas Department of State Health Services (DSHS). On November 27, Houston LRN reported to DSHS Region 8 (San Antonio, TX, USA) confirmation of the isolate from hospital A as *B. pseudomallei*. The Houston LRN and hospital A destroyed the remaining isolates because of regulations surrounding handling of select agents.

DSHS and CDC collaboratively investigated the source of the patient’s exposure to *B. pseudomallei* and performed risk assessments to identify potential laboratory exposures at both hospitals.

The patient’s only reported travel outside Texas was a visit to Monterrey, Mexico, 30 years before illness onset. Before becoming ill, he resided on a small rural ranch without running water from a municipal source or private well. He purchased water for nondrinking use from a local chlorinated municipal water utility. He used a 500-gallon uncovered tank to store the water and then pumped the water into a 1,600-gallon storage tank. He cleaned the tank 1–2 times/month by climbing inside to scrub the walls. The last cleaning was 2 days before illness onset.

During environmental sampling in December 2018, we collected 56 specimens from the patient’s home and property. We concentrated large-volume (30–190 L) water samples on site and processed as described (*8*). We collected soil samples (50 mL) at a depth of 30 cm (*9*) from 8 sites in moist soil. Other environmental samples included surface swabs (10 × 10 inch) of the inside of both water storage tanks and swabs of indoor and outdoor plumbing fixtures. We processed specimens by using international guidelines (*9*) and tested for presence of *B. pseudomallei* by using culture and real-time quantitative PCR (*10*). All specimens were negative for *B. pseudomallei*.

We identified 7 laboratory personnel at hospitals A and B who had low-risk exposures to the *B. pseudomallei* isolates (*5*). Recommendations included temperature monitoring twice a day for 21 days and collecting serum samples at 1, 2, 4 and 6 weeks postexposure (*5*). We performed serologic testing by using an indirect hemagglutination assay (*11*). All samples were negative for *B. pseudomallei* antibodies (cutoff value 1:40).

**Figure 2:**
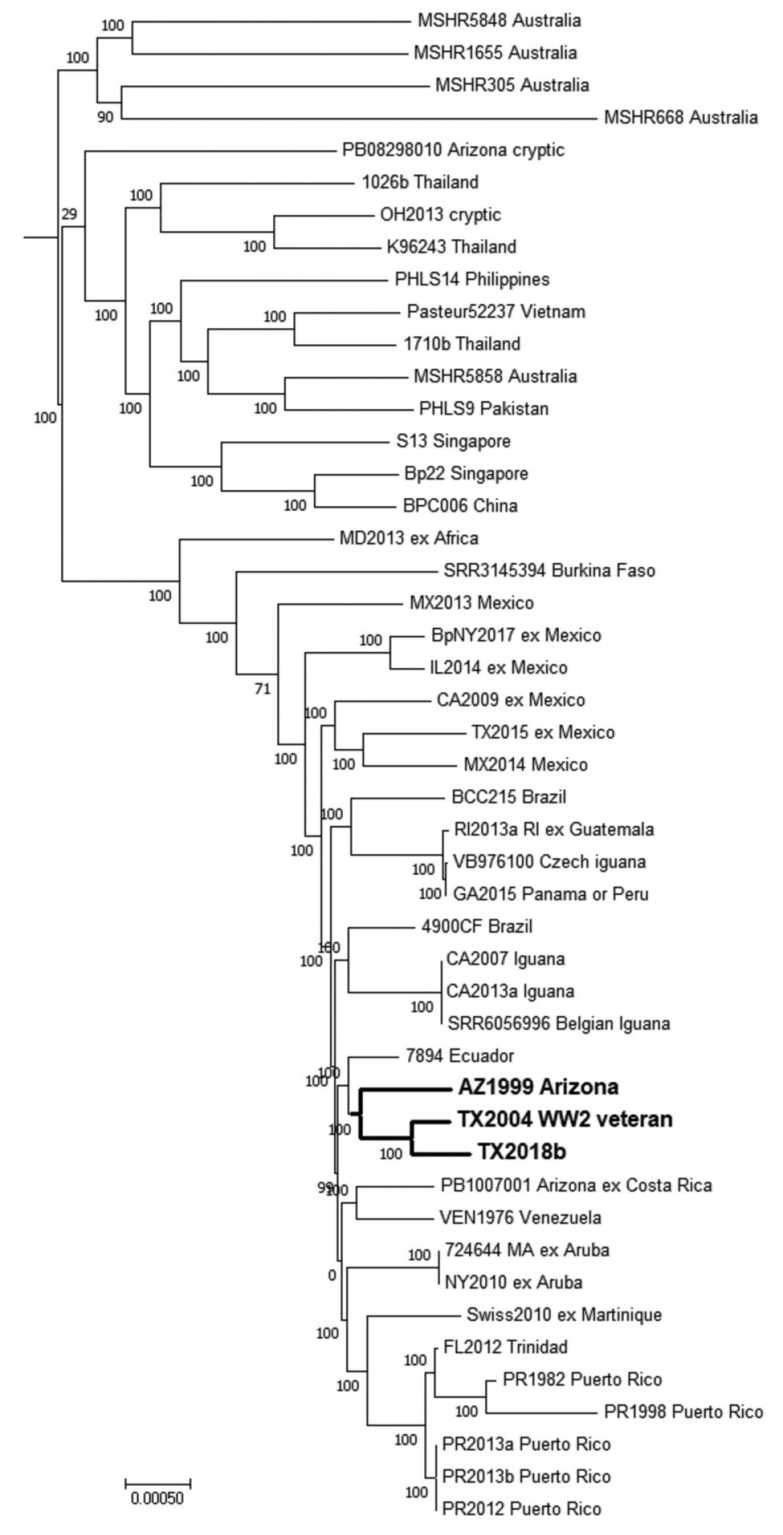
Dendrogram for characterization of *Burkholderia pseudomallei* isolate TX2018b from a 63-year-old man in Texas, USA, by comparison with reference genomes. Maximum-parsimony phylogenetic analysis based on core single-nucleotide polymorphisms (SNP) by using Parsnp, a component of the Harvest 1.3 software (https://github.com/marbl/harvest). Bold indicates clusters of genomes associated with the southwestern United States; the 2004 patient resided in the same county as the patient described in this article. Numbers at each node are bootstrap percentages. Scale bar indicates nucleotide substitutions per SNP.

Hospital B submitted the patient’s blood culture isolate (TX2018b) for whole-genome sequencing (National Center for Biotechnology BioProject no. PRJNA545616). Multilocus sequence typing identified the isolate as sequence type 297 (*12*). The 8 other sequence type 297 isolates are all associated with North America (*1*). A higher resolution single-nucleotide polymorphism comparison of the draft genome assembly with other public assemblies containing geographic data also indicated that the isolate is associated with others from North America. TX2018b grouped closest to TX2004 and to a lesser extent to AZ1999 (BioProject no. PRJNA545640) (Figure 2).

TX2004 was isolated in 2004 from a patient residing in the same Texas county as our patient (*13*). It was originally hypothesized that the 2004 patient was infected 62 years before disease onset, while serving during World War II in Southeast Asia (*13*). However, TX2004 is not related to strains from Southeast Asia but to strains from the Americas (*1*). Other regional travel by the 2004 patient was not reported. AZ1999 was isolated from a patient treated in 1999 in Arizona who had recently emigrated from El Salvador. Where exposure occurred for that patient is unknown, but that isolate groups closer to TX2004 and TX2018b than to other examples associated with Central America (Figure 2).

## Conclusions

The source of this patient’s infection remains unknown. However, genomic analysis showed that the patient isolate groups with existing isolates collected from other patients in the southwestern United States. Isolates TX2004 and TX2018b were collected ≈15 years apart from patients living in the same Texas county at time of illness onset and group together, a finding that suggests *B. pseudomallei* might be present in the environment in this area. Furthermore, these 2 isolates might represent a new clade endemic to the continental United States. Further investigation is warranted because this region is predicted to have suitable habitats for *B. pseudomallei* (*14*).

These findings call into question possible reactivation of melioidosis decades after travel to melioidosis-endemic regions. Instead of a 62-year incubation period, the patient infected with TX2004 might have had an unknown local environmental exposure that preceded symptom onset. A 1991 case report of an 18-year latency for a Vietnam War veteran indicated that this patient was also living in the southwestern United States (New Mexico) at time of symptom onset and had not traveled outside the continental United States since 1971 (*15*). On the other hand, although geographic and genotypic links between the 2 Texas cases suggest a local source, *B. pseudomallei* exposure for the patient infected with TX2018b could have occurred 30 years earlier, while visiting Mexico, and the patient infected with TX2004 might have had unreported regional travel before illness onset. Only when *B. pseudomallei* is isolated from the environment can it be definitively stated that *B. pseudomallei* is endemic to the continental United States.

*B. pseudomallei* infection should be included in a differential diagnosis for a patient with compatible disease, even without reported travel history. Increased awareness among healthcare workers and diagnostic laboratory personnel for melioidosis as a disease potentially endemic to the southwestern United States is critical to improve case outcomes and prevent laboratory exposures.

In addition, melioidosis is caused by a Tier 1 overlap Select Agent, but reporting of cases to CDC is voluntary (*2*,*7*). Making melioidosis nationally notifiable could improve surveillance and recognition and clarify distribution and potential sources of *B. pseudomallei* infection in the United States.
